# The atherogenic index of plasma plays an important role in predicting the prognosis of type 2 diabetic subjects undergoing percutaneous coronary intervention: results from an observational cohort study in China

**DOI:** 10.1186/s12933-020-0989-8

**Published:** 2020-02-21

**Authors:** Zheng Qin, Kuo Zhou, Yueping Li, Wanjun Cheng, Zhijian Wang, Jianlong Wang, Fei Gao, Lixia Yang, Yingkai Xu, Yafeng Wu, Hua He, Yujie Zhou

**Affiliations:** 1 Department of Cardiology, Beijing Anzhen Hospital, Capital Medical University, Beijing, 100029 China; 2 Department of Emergency Cardiology, Beijing Anzhen Hospital, Capital Medical University, Beijing, 100029 China; 3 Department of Cardiology, Beijing Anzhen Hospital, Beijing Institute of Heart Lung and Blood Vessel Disease, Beijing Key Laboratory of Precision Medicine of Coronary Atherosclerotlic Disease, Clinical Center for Coronary Heart Disease, Capital Medical University, No. 2 Anzhen Road, Chaoyang District, Beijing, 100029 China

**Keywords:** Atherogenic index of plasma, Type 2 diabetes mellitus, Major cardiovascular and cerebrovascular adverse events, Percutaneous coronary intervention

## Abstract

**Background:**

Many studies have reported the predictive value of the atherogenic index of plasma (AIP) in the progression of atherosclerosis and the prognosis of percutaneous coronary intervention (PCI). However, the utility of the AIP for prediction is unknown after PCI among type 2 diabetes mellitus (T2DM).

**Methods:**

2356 patients with T2DM who underwent PCI were enrolled and followed up for 4 years. The primary outcome was major cardiovascular and cerebrovascular adverse events (MACCEs), considered to be a combination of cardiogenic death, myocardial infarction, repeated revascularization, and stroke. Secondary endpoints included all-cause mortality, target vessel revascularization (TVR), and non-target vessel revascularization (non-TVR). Multivariate Cox proportional hazards regression modelling found that the AIP was correlated with prognosis and verified by multiple models. According to the optimal cut-off point of the ROC curve, the population was divided into high/low-AIP groups. A total of 821 pairs were successfully matched using propensity score matching. Then, survival analysis was performed on both groups.

**Results:**

The overall incidence of MACCEs was 20.50% during a median of 47.50 months of follow-up. The multivariate Cox proportional hazards regression analysis before matching suggested that the AIP was an independent risk factor for the prognosis of T2DM after PCI (hazard ratio [HR] 1.528, 95% CI 1.100–2.123, P = 0.011). According to the survival analysis of the matched population, the prognosis of the high AIP group was significantly worse than that of the low AIP group (HR (95% CI) 1.614 (1.303–2.001), P < 0.001), and the difference was mainly caused by repeat revascularization. The low-density lipoprotein-cholesterol (LDL-C) level did not affect the prognosis of patients with T2DM (P = 0.169), and the effect of the AIP on prognosis was also not affected by LDL-C level (P < 0.001).

**Conclusions:**

The AIP, a comprehensive index of lipid management in patients with T2DM, affects prognosis after PCI. The prognosis of diabetic patients with high levels of the AIP included more MACCEs and was not affected by LDL-C levels. It is recommended to monitor the AIP for lipid management in diabetic patients after PCI and ensure that the AIP is not higher than 0.318.

*Trial registration* This is an observational cohort study that does not involve interventions. So we didn’t register. We guarantee that the research is authentic and reliable, and hope that your journal can give us a chance.

## Background

Cardiovascular disease is one of the leading causes of death in patients with type 2 diabetes mellitus (T2DM) [1]. Despite adequate attention from clinicians, its prognosis in T2DM patients is still significantly worse than that in non-diabetic patients [2]. Abnormal lipid metabolism, including high triglyceride (TG), low high-density lipoprotein-cholesterol (HDL-C), and high low-density lipoprotein-cholesterol (LDL-C) levels and abnormal glucose metabolism (insulin resistance), is commonly present in diabetic patients and makes significant contributions to poor prognosis [3]. The atherogenic index of plasma (AIP) is calculated by log (TG/HDL-C) [4], which can reflect the characteristics of abnormal lipid metabolism in diabetic patients and can quantify the degree of abnormal lipid metabolism. In addition, the AIP is correlated with the degree of insulin resistance [5], which can also indicate the degree of abnormal glucose metabolism.

Studies [6] have shown that the AIP has predictive value for atherosclerosis, which indicates a significant positive correlation between diabetes mellitus and the AIP, as well as between carotid intima-media thickness (cIMT) progression and arterial stiffness. Furthermore, elevated AIP is a powerful independent predictor of all-cause mortality and subsequent cardiovascular disease after coronary revascularization [7]. In subpopulations of patients with diabetes, the AIP is related to the incidence of type 2 diabetes [8], while some studies have suggested that the AIP is a risk factor for coronary heart disease in patients with type 2 diabetes [9, 10]. However, whether the AIP still plays a role after percutaneous coronary intervention (PCI) in diabetic patients is still unknown. Therefore, we investigated the relationship between the AIP and long-term follow-up outcomes after PCI in patients with type 2 diabetes.

## Methods

### Patient population and study design

**Fig. 1:**
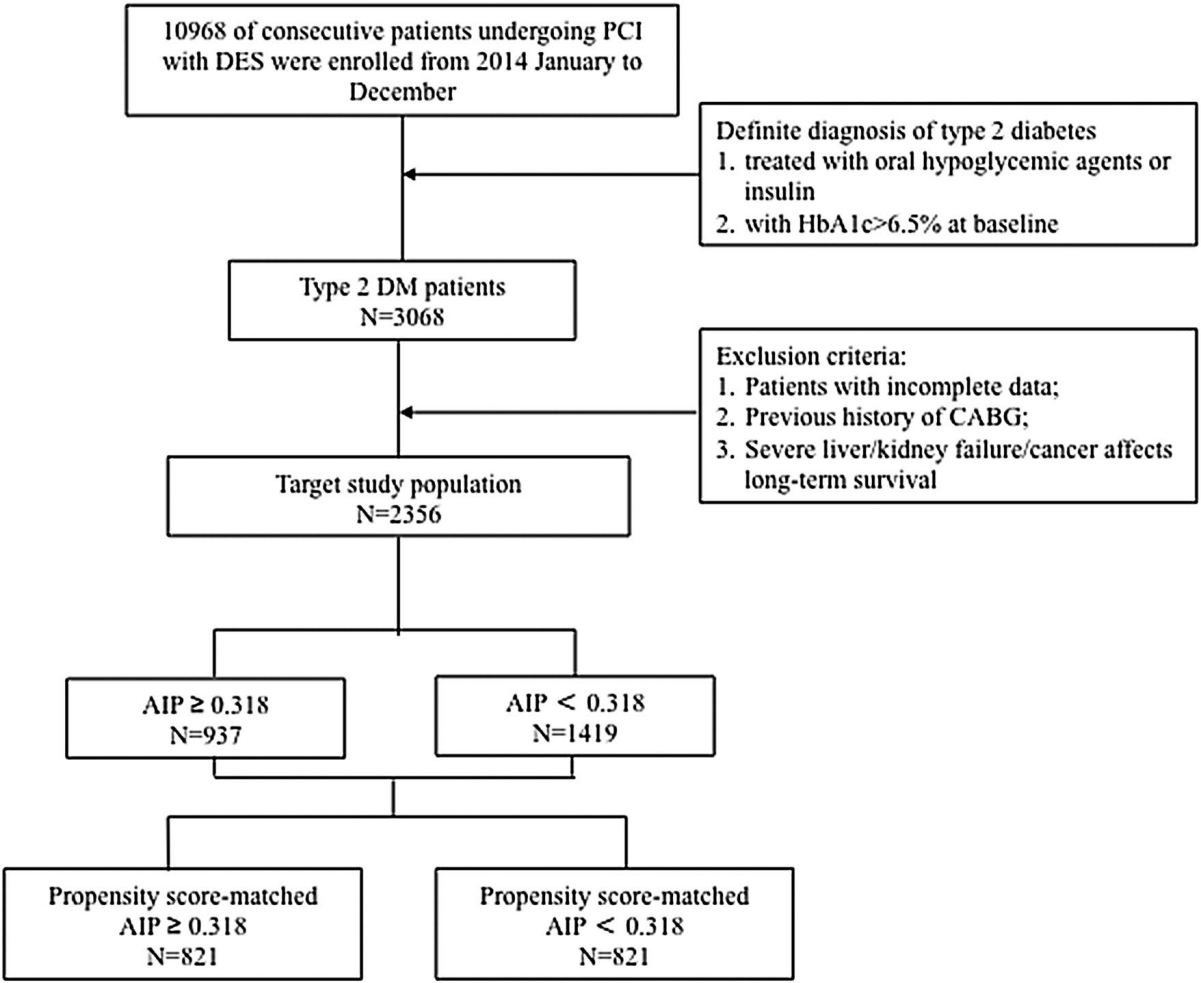
Flow chart of enrolled patients

The present study is a large single-centre observational cohort study that mainly occurred in Beijing Anzhen Hospital, Capital Medical University (one of the largest interventional diagnostic and treatment centres in the world, with approximately 40,000 interventional diagnostic and treatment cases annually). A total of 10 968 consecutive patients underwent PCI with drug-eluting stents (DES) in our hospital from January to December 2014. Of those, 3068 patients with clearly diagnosed type 2 diabetes were enrolled. The exclusion criteria were as follow: patients with incomplete baseline and follow-up data; those with a previous history of CABG; or those with severe liver/kidney failure, cancer or other major diseases that affect long-term survival. Ultimately, 2356 patients were included in the statistical analysis. Patients underwent follow-up via telephone or outpatient service after the first 1/6/12 months after PCI and annually thereafter. All patients were followed-up for 4 years unless death occurred. Multivariate Cox proportional hazards regression modelling indicated that the AIP was correlated with prognosis and verified by multiple models. The optimal cut-off point of the AIP was found (AIP = 0.318) with the ROC curve, and the patients were divided into a high AIP group (AIP ≥ 0.318) and a low AIP group (AIP < 0.318). Patients in the two groups were matched according to the 1:1 propensity score, and 821 pairs of patients were successfully matched for survival analysis (see Fig. 1 for the detailed process). This study was approved by the Clinical Research Ethics Committee of Beijing Anzhen Hospital, Capital Medical University, and all patients were informed and agreed to participate in this study.

### Data collection

Data collection was mainly conducted by case report form (CRF), which mainly included patients’ demographic and clinical characteristics, age, sex, previous medical history, physical examination, laboratory examination, intervention-related data, types of drugs taken after PCI, follow-up information, etc. Blood samples were collected the next morning after an overnight fast. Lipid-related blood tests were performed for patients taking statins for more than 1 month. The calculation of the AIP is log (TG/HDL-C) on an empty stomach. The SYNTAX score of each patient was calculated using the online scoring system (www.syntaxscore.com) by scoring all coronary artery lesions with diameters ≥ 50% in vessels with reference diameters ≥ 1.5 mm [11].

Coronary angiography data at baseline and follow-up, such as the number of target vessels, minimum stent diameter, average stent length, and SYNTAX, were performed separately by two experienced interventional physicians. Follow-up was performed independently by 2 trained medical personnel (who were blinded to patient information).

### Clinical end points and definitions

The primary outcome was major cardiovascular and cerebrovascular adverse events (MACCEs), considered to be a combination of cardiogenic death, myocardial infarction, repeated revascularization, and stroke. Secondary endpoints included all-cause mortality, target vessel revascularization (TVR), and non-target vessel revascularization (non-TVR). MACCEs were considered the first occurrence of an event during each patient’s follow-up. Cardiac death, myocardial infarction, repeat revascularization, and stroke were all events recorded for each patient during the 4-year follow-up period.

Diabetic patients were defined as patients who had a previous diagnosis of DM (treated with diet, oral agents or insulin) or a new diagnosis of DM (FBG ≥ 7.0 mmol/L on 2 occasions during hospitalization, HbA1c > 6.5% at baseline) based on the current guidelines [12]. Hypertension was defined by systolic blood pressure (SBP) ≥ 140 mmHg and/or diastolic blood pressure (DBP) ≥ 90 mmHg and/or the use of antihypertensive treatment in the past 2 weeks [13]. Hyperlipidaemia was defined by an increase in plasma TG or TC but also included an increase in LDL cholesterol and a decrease in HDL cholesterol [14]. Adult LDL-C ranged from 2.07 to 3.37 mmol/L (80 to 130 mg/day). Smoking was defined as smoking at the time of PCI or having quit smoking for less than 1 year; target vessel revascularization was defined as any revascularization procedure, either percutaneous or surgical, involving the target vessel.

### Statistical analysis

We performed Tests of Normality with Kolmogorov–Smirnov and Shapiro–Wilk to test. Continuous variables with a normal distribution, those with a non-normal distribution and categorical variables were expressed as the mean ± standard deviation, median/quartile and counts or percentages, respectively, and differences were detected with a t-test of two independent samples, rank sum test and Chi-square test, respectively. The incidence of events is expressed in terms of the number of incidents during the 4-year follow-up. Cox proportional hazards regression modelling was used to analyse independent risk factors associated with MACCE. The prognostic risk factors of each model adjustment include: Model: adjusted analysis. Model2: adjusted on Age, Sex, BMI, SBP, DBP, Smoking, drinking, DM time, hypertension, hyperlipidemia, stroke, MI, history of CAD, PCI, stable angina pectoris, UA/NSTEMI, STEMI. Model3: adjusted on model2 + HBC1, CREA, LVEF, TC, and LDL. Model4: adjusted on model3 + asprin, clopidigrel, b-blocker, statin, ACEI/ARB, oral antidiabetic agents, insulin. The ROC curve determined the optimal cut-off point of the AIP. Propensity score-matched analysis was used to conduct 1:1 matching between high AIP and low AIP. The Kaplan–Meier method was used for graphical evaluation of time-related events and was evaluated by log-rank tests. Statistical significance was accepted at the 95% confidence level (CI) (two-sided P ≤ 0.05). SPSS software for Windows (version 24.0, SPSS Inc., Chicago, Illinois) was used for statistical analyses.

## Results

### Differences in baseline clinical and angiographic characteristics among the MACE and non-MACE groups of the study population (total population)

The baseline clinical and angiographic characteristics of the total population are shown in Table 1.

**Table 1:** Difference in baseline clinical and angiographic characteristics among the MACE and non-MACE group of study population (total population)

Characteristics	MACE (n = 483)	Non-MACE (n = 1873)	P value
Demographic			
Age, years	58.03 ± 9.039	57.95 ± 9.182	0.867
Male, n (%)	353 (73.1)	1385 (73.9)	0.701
Behavioral			
Smoking, n (%)	215 (44.5)	832 (44.4)	0.971
Drinking, n (%)	85 (17.6)	331 (17.7)	0.97
Physical			
BMI, kg/m^2^	26.07 ± 3.564	26.07 ± 3.504	0.985
SBP, mmHg	129.64 ± 14.982	130.5 ± 16.485	0.27
DBP, mmHg	77.67 ± 9.913	77.8 ± 10.614	0.81
Medical history, n (%)			
Hypertension	299 (61.9)	1208 (64.5)	0.29
Hyperlipidemia	209 (43.3)	812 (43.4)	0.974
History of MI	50 (10.4)	201 (10.7)	0.81
History of stroke	29 (6.0)	144 (7.7)	0.206
Family history of CAD	62 (12.8)	260 (13.9)	0.551
Previous PCI	79 (16.4)	317 (16.9)	0.766
Diagnosed DM, years	7.82 ± 3.73	6.58 ± 3.942	< 0.001
Clinical presentation, n (%)			
Stable CAD	52 (10.8)	249 (13.3)	0.138
Unstable angina/NSTEMI	352 (72.9)	1267 (67.6)	0.027
STEMI	79 (16.3)	357 (19.1)	0.172
Medical treatment, n (%)			
Asprin	479 (99.2)	1862 (99.4)	0.785
Clopidogrel	476 (98)	1832 (97.8)	0.305
β-Blocker	391 (81)	1498 (80)	0.632
Statins	473 (97.9)	1827 (97.5)	0.62
ACEI/ARB	275 (56.9)	1028 (54.9)	0.419
Oral hypoglycemic drugs	353 (72.9)	1322 (70.6)	0.321
Insulin	183 (37.9)	649 (34.7)	0.184
Laboratory results			
TC, mmol/L	4.25 ± 1.111	4.11 ± 1.091	0.015
LDL-C, mmol/L	2.49 ± 0.841	2.47 ± 0.89	0.643
AIP	0.3 ± 0.356	0.22 ± 0.292	< 0.001
HbA1c, %	8.1 ± 1.269	7.4 ± 1.299	< 0.001
hs-CRP, mg/L	4.18 ± 6.708	4.01 ± 6.877	0.614
Creatinine, μmol/L	71.98 ± 17.595	71.46 ± 19.905	0.604
GFR, mL/min	95.13 ± 20.680	95.79 ± 19.612	0.515
LVEF, %	61.44 ± 8.231	62.09 ± 8.15	0.118
Number of target vessels			0.229
One, n (%)	206 (45.1)	856 (45.7)	
Multivessel, n (%)	277 (57.3)	1017 (54.3)	
Target vessels			
LM, n (%)	17 (3.5)	75 (4)	0.624
LAD, n (%)	290 (60)	1121 (59.9)	0.939
LCX, n (%)	720 (34.8)	200 (41.4)	0.233
RCA, n (%)	212 (43.9)	782 (41.8)	0.396
SYNTAX score	14.61 ± 6.971	12.48 ± 7.162	< 0.001
Minimal stent diameter, mm	2.92 ± 0.457	2.92 ± 0.46	0.931
Average stent length, mm	22.27 ± 6.748	22.16 ± 6.43	0.749
Types of stent, n (%)			0.338
First generation DES	225 (46.6)	827 (44.2)	
Second generation DES	258 (53.4)	1046 (55.8)	

Continuous variables were expressed as mean (${\bar{\text{x}}}$) ± standard deviation (SD) in case of normal distribution and compared between two groups by two-independent samples *t*-test. Data were expressed as medians (interquartile ranges) in case of skewed distribution and compared using the Mann–Whitney *U*-test. Categorical variables are presented as counts (percentages) and compared by Chi-square test

*MACCE* major adverse cardiac and cerebrovascular event, *BMI* body mass index, *SBP* systolic blood pressure, *DBP* diastolic blood pressure, *DM* diabetes mellitus, *MI* myocardial infraction, *CAD* coronary artery disease, *PCI* percutaneous coronary intervention, *NSTEMI* non-ST-segment–elevation myocardial infarction, *STEMI* ST-segment–elevation myocardial infarction, *TG* triglyceride, *TC* total cholesterol, *LDL-C* low density lipoprotein cholesterol, *HDL-C* high density lipoprotein cholesterol, *AIP* atherogenic index of plasma, *HbA1c* glycosylated hemoglobin, *hs-CRP* high-sensitivity C-reactive protein, *LVEF* left ventricular ejection fraction, *ACEI* angiotensin converting enzyme inhibitor, *ARB* angiotensin receptor blocker, *LM* left main, *LAD* left anterior descending, *LCX* left circumflex artery, *RCA* right coronary artery, *SYNTAX* synergy between PCI with taxus and cardiac surgery, *DES* drug-eluting stent

There was no significant difference between the MACCE group and the non-MACCE group in age, sex, medical history, or medical treatment. There were significant differences in the duration of diabetes, HbA1c, TG, TC, HDL-C, the AIP, and SYNTAX score. In Cox proportional hazards regression modelling, the AIP was one of the independent predictors of prognosis in patients with diabetes after adjusting for other confounding factors, and multiple models were used for verification (Table 2). ROC curve analysis showed that the AIP had good predictive accuracy for prognosis. Baseline AIP was identified at 0.318 as the optimal cut-off point to predict the risk of prognosis.

**Table 2:** Independent predictors of MACCES in patients with DM after baseline PCI

Variables	HR	95% CI	*P* values
Model1	2.137	1.600–2.856	< 0.001
Model2	1.619	1.170–2.241	0.004
Model3	1.618	1.169–2.239	0.004
Model4	1.528	1.100–2.123	0.011

Model1: age, male, BMI, SBP, DBP, smoking, drinking, diagnosed DM, hypertension, hyperlipidemia, history of MI, history of stroke, family history of CAD, previous PCI, clinical presentation

Model2: Model1 + HbA1c, CRP, CREA, LVEF, TC-C, LDL-C

Model3: Model2 + medical treatment (aspirin, clopidogrel, β-blocker, statins, ACEI/ARB, oral hypoglycemic drugs, insulin)

Model4: Model3 + number of target vessels, target vessels (LM, LAD, LCX, RCA), minimal stent diameter, average stent length, types of stent, SYNTAX score

### Baseline clinical and angiographic characteristics and Kaplan–Meier curve (propensity score-matched population)

**Fig. 2:**
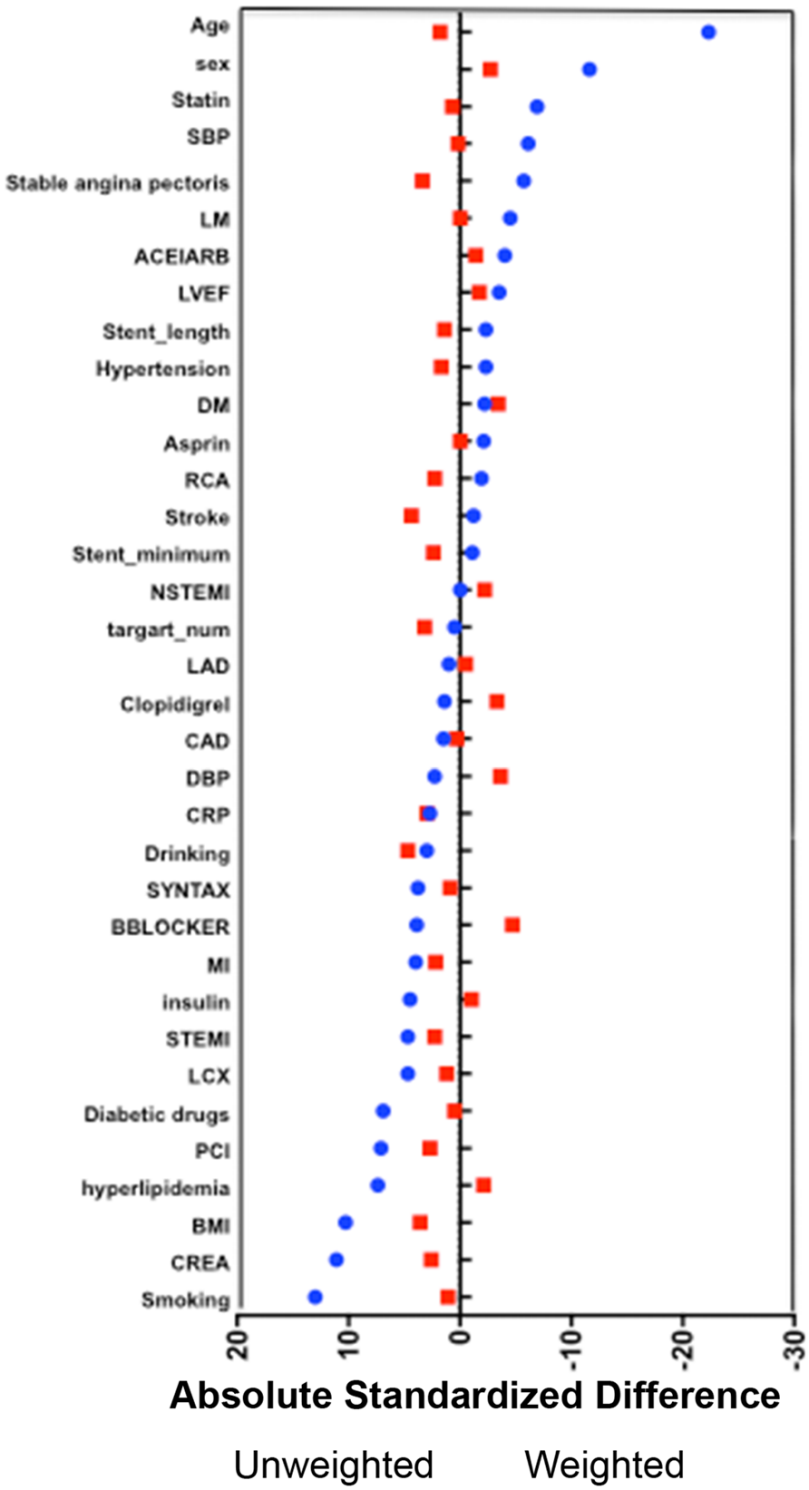
The baseline clinical and angiographic characteristics before and after propensity score-matched. *HDL-C* high density lipoprotein cholesterol, *ARB* angiotensin receptor blocker, *LDL-C* low density lipoprotein cholesterol, *ACEI* angiotensin converting enzyme inhibitor, *LAD* left anterior descending, *SYNTAX* synergy between PCI with taxus and cardiac surgery, *LVEF* left ventricular ejection fraction, *DBP* diastolic blood pressure, AIP atherogenic index of plasma

**Fig. 3:**
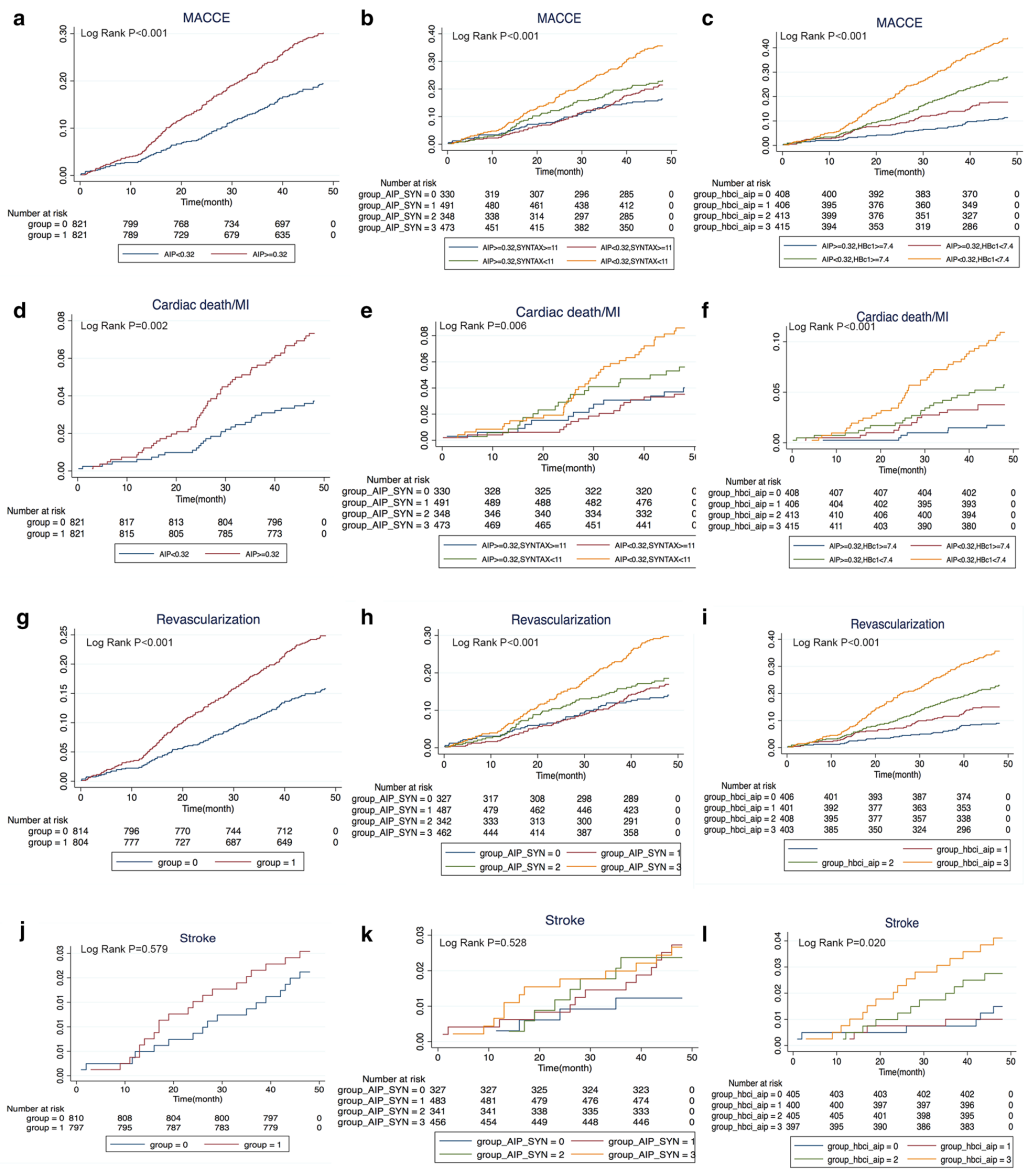
Comparison of estimated event rates in the propensity score-matched population. Kaplan–Meier curve of the matched population for overall survival analysis, and the influence of high/low AIP group on prognosis was evaluated, as well as combined the influence of SYNTAX score and HbA1c on prognosis, respectively. **a**–**c** MACCE rates, **d**–**f** cardiac death/MI rates, **g**–**i** repeat revascularization rate, **j**–**l** Stroke rates. Target incidence of observation considering AIP level and SYNTAX score (**b**, **e**, **h**, **k**) or HbA1c (**c**, **f**, **i**, **l**). *MACCE* major adverse cardiac and cerebrovascular events, *AIP* atherogenic index of plasma, *SYNTAX* synergy between PCI with taxus and cardiac surgery, *HbA1c* glycosylated hemoglobin, *PCI* percutaneous coronary intervention

**Fig. 4:**
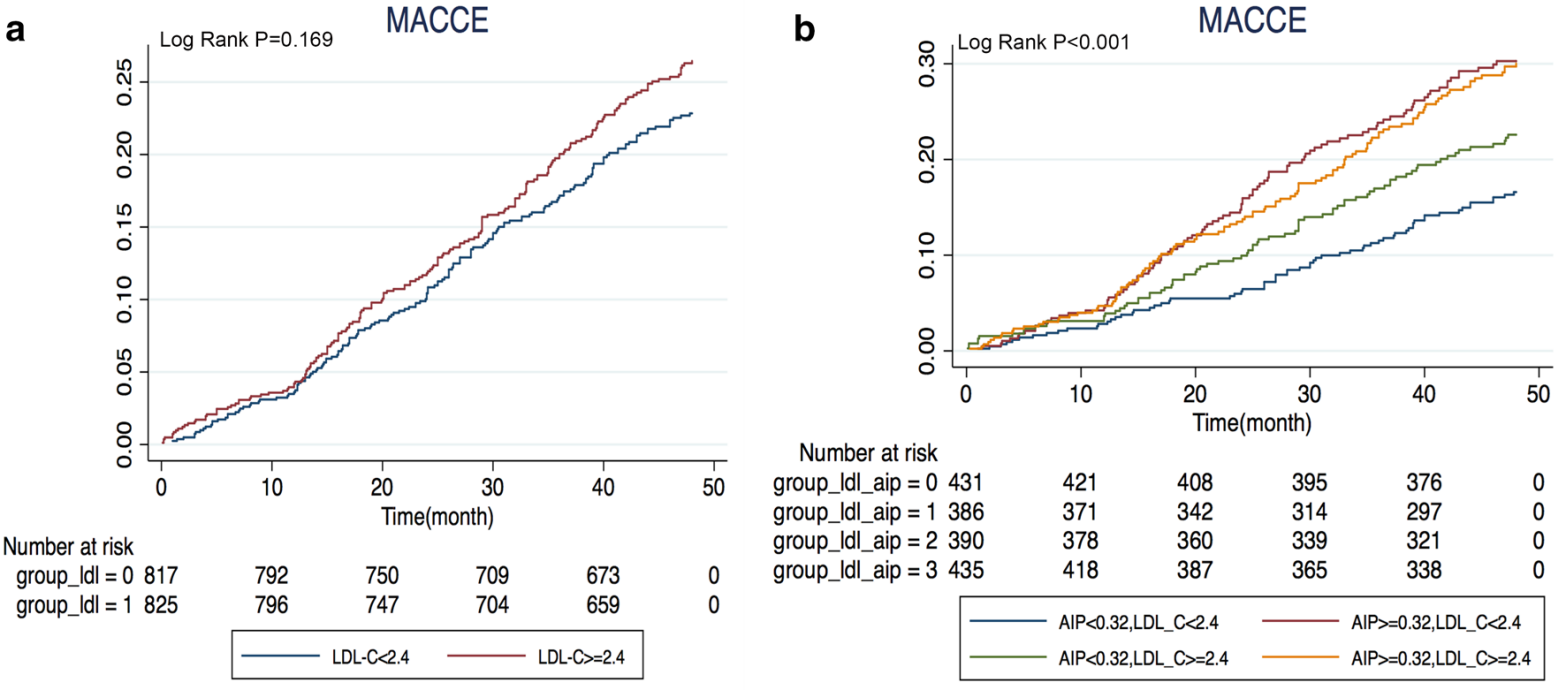
Comparison of estimated event rates in the propensity score-matched population. **a** Comparison of MACCE rate considering LDL-C level; **b** comparison of MACCE rate considering LDL-C and AIP level. *MACCE* major adverse cardiac and cerebrovascular events, *LDL-C* low-density lipoprotein cholesterol, *AIP* atherogenic index of plasma, *PCI* percutaneous coronary intervention

The clinical baseline characteristics before and after propensity score matching are shown in Fig. 2. There was no significant difference between the low AIP group and the high AIP group in age, sex, BMI, medical history, duration of diabetes, HbA1c, TC, LDL-C, drug use, other biomarkers, angiography and process characteristics. The C statistic value of the propensity score model was 0.01. We tabulated the events that occurred in matched pre/post populations according to MACCE/all-cause mortality/cardiogenic death/myocardial infarction/stroke, etc. (see Table 3 for details). The log-rank test was used to compare the Kaplan–Meier curve of the matched population for overall survival analysis, and the influence of high/low AIP group on prognosis was evaluated, as well as the influence of a combination of the SYNTAX score and HbA1c on prognosis (see Fig. 3). In addition, given that LDL-C is a traditional classic risk factor for arteriosclerosis, the population was divided into high/low LDL-C groups to assess the influences on the MACCE rate and on prognosis under the influence of high/low AIP (see Fig. 4).

**Table 3:** Estimated Kaplan–Meier events rates of 4-year follow-up

Adverse events	Overall population				Propensity score-matched population			
	High AIP (n = 937)	Low AIP (n = 1419)	Adjusted HR^a^ (95% CI)	P Value	High AIP (n = 821)	Low AIP (n = 821)	HR (95% CI)	P value
MACCE	253 (27.0)	230 (16.2)	1.638 (1.363–1.969)	< 0.001	214 (26.1)	145 (17.7)	1.614 (1.303–2.001)	< 0.001
All-cause death	40 (4.3)	33 (2.3)	1.690 (1.049–2.724)	0.031	34 (4.1)	21 (2.6)	1.750 (1.003–3.054)	0.049
Cardiac death	28 (3.0)	19 (1.3)	2.184 (1.197–3.984)	0.011	24 (2.9)	11 (1.3)	2.615 (1.251–5.470)	0.011
Cardiac death/MI	71 (7.58)	50 (3.52)	2.199 (1.531–3.158)	< 0.001	58 (7.06)	30 (3.65)	1.968 (1.266–3.058)	0.003
MI	58 (6.3)	41 (2.9)	1.962 (1.301–2.960)	0.001	47 (5.8)	25 (3.1)	2.080 (1.267–3.415)	0.004
Repeat revascularization	210 (22.9)	190 (13.5)	1.644 (1.343–2.013)	< 0.001	177 (22.0)	119 (14.6)	1.628 (1.285–2.062)	< 0.001
TVR	125 (13.6)	99 (7.0)	1.768 (1.347–2.322)	< 0.001	105 (13.1)	60 (7.4)	1.809 (1.310–2.498)	< 0.001
Non-TVR	85 (9.4)	91 (6.5)	1.413 (1.043–1.916)	0.026	72 (9.0)	59 (7.3)	1.314 (0.927–1.865)	0.125
Stroke	23 (2.5)	27 (1.9)	1.124 (0.628–2.014)	0.694	20 (2.5)	17 (2.1)	1.191 (0.610–2.325)	0.608

Values expressed are n (%) or hazard ratio (95% confidence interval). The percentages shown are Kaplan–Meier estimates from the intention to treat analysis

Major adverse cardiac and cerebrovascular events included cardiac death, myocardial infarction, repeat revascularization, and stroke

*AIP* atherogenic index of plasma, *CI* indicates confidence interval, *HR* hazard ratio, *MACCE* major adverse cardiac and cerebrovascular event, *MI* myocardial infarction, *TVR* target vessel revascularization

^a^Hazard ratio was adjusted with age, body mass index, sex, medical history, medical treatment

## Discussion

### Main findings in this study

In the present single-centre observational study, we found that the AIP, a parameter related to abnormal lipid and glucose metabolism, plays an important role in the long-term prognosis of type 2 diabetes patients undergoing percutaneous coronary intervention. MACCEs were significantly reduced in the group with lower AIP after PCI compared to the group with higher AIP. This benefit was mainly due to a reduction in the rate of repeated revascularization. In the subgroup analysis, we found that the effect of the AIP on prognosis was independent of LDL-C level.

### Patients with T2DM have a poor prognosis, which is mainly related to abnormal metabolism

The mechanism of atherosclerosis is complex, and epidemiological studies suggest that the internationally recognized risk factors for coronary heart disease include dyslipidaemia, hypertension, diabetes and smoking [15]. In type 2 diabetes, atherosclerosis has the same mechanism—lipids and inflammation can also cause damage to cardiovascular organs [16]. There is a direct association between inflammatory cytokines, interleukins and matrix-metalloproteinase 12 (MMP-12), as well as an inverse association between MMP-12 and HDL, both in T2DM patients and in non-T2DM subjects. However, patients with type 2 diabetes also have a unique mechanism of atherosclerosis. For example, in patients with elevated total cholesterol, LDL-C or non-HDL-C, an increase in LP(a) can lead to additional adverse events in patients with T2DM [17]. Abnormal lipid metabolism and abnormal glucose metabolism are present in T2DM patients. Under the combined influence of these two factors, the lesions are more severe, and the prognosis is worse [18]. Therefore, the management of blood lipids and glucose after PCI is of great significance for the prognosis of diabetic patients.

### The AIP-A comprehensive index of specific dyslipidaemia in diabetic patients

At present, a single lipid index cannot fully reflect cardiovascular disease. The triglyceride-glucose index is positively associated with a higher prevalence of symptomatic CAD and metabolic and behavioural risk factors and could also be used as a marker for atherosclerosis [19]. Similarly, the AIP, calculated by log (TG/HDL-C), which is a comprehensive index of blood lipids [20], is almost always associated with other CVD risk factors [21], and a higher AIP value is positively and strongly associated with obesity [22]. Hyperlipidaemia is closely related to atherosclerosis, of which LDL-C is the most important component. However, there was no significant difference in LDL-C levels between diabetic and non-diabetic patients in previous studies on the prognosis of PCI [23]. In the prognostic analysis of the diabetes subgroup, LDL-C levels were also not increased in patients with poor prognosis [4]. Therefore, the classic single lipid index (LDL-C) can no longer explain the severe lesions and poor prognosis in diabetic patients. Abnormal lipid metabolism in diabetic patients is often manifested as high TG and low HDL-C, while TC and LDL-C are usually normal or slightly elevated [3].

LDL-C is the core component of atherosclerosis in blood lipids. However, LDL-C is mostly normal in diabetic patients with abnormal lipid metabolism, which cannot explain the theory of atherosclerosis caused by blood lipids. Currently, sLDL-C, which is a subcomponent of LDL-C, does not easily bind to LDL receptors in circulation, reducing the clearance rate. Moreover, sLDL-C is easily oxidized and thus engulfed by macrophages to form foam cells, which then disintegrate and release a large amount of cholesterol [24]. sLDL-C is a more important and sensitive indicator of atherosclerosis [25]. The average diameter of LDL-C can indirectly reflect the number of sLDL-C particles, but it is not widely available in the clinic due to the complexity and high cost of measurement. Studies have shown that the AIP is negatively correlated with the particle size of LDL [3, 26]. To some extent, the AIP can be used to replace the number of sLDL-C particles.

Previous studies [27] have also confirmed that the AIP is more suggestive than LDL-C in atherosclerosis. In diabetic patients with normal LDL-C, the AIP can better predict the degree of atherosclerosis than LDL [28]. All the above evidence confirms that the AIP can comprehensively represent blood lipids in diabetic patients. In this study, the AIP also played an important role in predicting prognosis. MACCEs were significantly increased in patients with an AIP greater than 0.318 and were not affected by LDL-C levels. The AIP is an independent indicator that can reflect the comprehensive situation of blood lipids in diabetic patients. We believe that our study provides a new indicator for the management of blood lipids in diabetic patients after PCI.

### The AIP is also correlated with abnormal glucose metabolism

Previous studies [29, 30] confirmed that blood glucose management in patients with diabetes after PCI is very important, and MACCEs were significantly reduced in patients with strict blood glucose control. However, a few studies [31] refuted the prognostic benefits of blood glucose management.

Theoretically, hyperglycaemia, insulin resistance and hyperinsulinaemia can all cause lipid metabolism disorders, oxidative stress and vascular endothelial damage, ultimately leading to the aggravation of coronary atherosclerosis [32]. Most patients with T2DM have insulin resistance, which gradually worsens. Insulin resistance reduces the activity of lipoprotein esterase, leading to a decrease in the clearance rate of very low-density lipoprotein, while an increase in hepatic lipase activity leads to the acceleration of the catabolism of high-density lipoprotein and eventually leads to abnormal lipid metabolism. Hyperinsulinaemia caused by insulin resistance can lead to the proliferation of smooth muscle and the formation of foam cells. These factors all accelerate hardening of the arteries. Blood insulin levels and insulin resistance reinforce each other in a vicious circle that eventually leads to hyperglycaemia. A hyperglycaemic environment can promote the production of early and late glycation products of the Amadori type, which leads to the development of vascular (coronary heart disease) and microvascular (diabetic nephropathy) diseases in patients with T2DM [33, 34].

Previous studies suggested that the AIP was negatively correlated with insulin sensitivity in diabetic patients [5]. Therefore, the use of the AIP in diabetic patients can not only describe the comprehensive situation of blood lipids but also reflect the degree of insulin resistance. In addition, propensity score matching was performed to adjust baseline characteristics and differences in subgroup analysis to verify that the AIP effects were consistent across subgroups. In addition, we adjusted for baseline characteristics between the two groups by propensity score matching to make the results more reliable.

In this study, we found a correlation between the AIP and clinical outcomes in diabetic patients. Low values of the AIP can significantly reduce MACCEs in diabetic patients after PCI, providing a new clinical indicator for secondary prevention of coronary heart disease in diabetic patients. Although the limitations of the study itself prevent us from finding accurate reference values of the AIP for clinical guidance, persistent management of blood lipids and blood glucose is a matter of constant attention.

### Limitations

Some limitations and strengths of the present study have to be acknowledged. First, this study was a single-centre observational study, not a randomized controlled trial. The severity of CHD was calculated based on the clinical manifestations of CHD (stable/unstable/NSTEMI/STEMI) and the SYNTAX score. Hypertension/hyperlipidaemia was classified by prior medical history, but there is no detailed breakdown of severity. Although the sample size was large and the propensity score was matched, there were still unforeseeable confounding factors affecting the experimental results. Second, we only included stable blood lipid levels after statin use and did not monitor the blood lipid levels 4 years after PCI. Repeated measurement of the AIP during follow-up may be of further value for prediction of MACCEs. Third, we monitored HbA1c while the patients were in the hospital. Although it can reflect the short-term glycaemic control of patients, it cannot represent the long-term glycaemic control of patients after PCI.

## Conclusions

The AIP is related to MACCEs after PCI in patients with T2DM: a low AIP can improve prognosis. Optimizing blood lipid and blood glucose management according to the value of the AIP is a new choice for secondary prevention of coronary heart disease in diabetic patients. More rigorous studies with larger sample sizes are expected in the future to verify our conclusions.

## Data Availability

The datasets generated and analysed for this study are available from the corresponding author upon reasonable request.

## Abbreviations

AIP: atherogenic index of plasma
ACEI: angiotensin-converting enzyme inhibitor
ARB: angiotensin receptor blocker
BMI: body mass index
BP-DESs: biodegradable polymer drug-eluting stent
CAD: coronary artery disease
cIMT: carotid intima-media thickness
CRF: case report form
DBP: diastolic blood pressure
DM: diabetes mellitus
ECG: electrocardiograph
FBG: fasting blood glucose
G2-DESs: second-generation drug-eluting stent
GFR: glomerular filtration rate
HDL-C: high-density lipoprotein cholesterol
HPLC: high-performance liquid chromatography
hs-CRP: high-sensitivity C-reactive protein
ISR: in-stent restenosis
LAD: left anterior descending
LCX: left circumflex artery
LDL-C: low-density lipoprotein cholesterol
LM: left main
LVEF: left ventricular ejection fraction
MI: myocardial infraction
MMP: matrix metalloproteinases
MVD: multivessel disease
non-HDL-C: non-high-density lipoprotein cholesterol
PCI: percutaneous coronary intervention
RCA: right coronary artery
ROC: receiver operating characteristics
RLP-C: remnant-like particle cholesterol
SBP: systolic blood pressure
SYNTAX: synergy between PCI with taxus and cardiac surgery
TC: total cholesterol
TG: triglyceride
UA: uric acid
